# Modified transanal opening of the intersphincteric space (TROPIS): a safe and effective procedure for transsphincteric fistula-in-ano in comparison with ligation of intersphincteric fistula tract (LIFT)

**DOI:** 10.1007/s10151-025-03193-5

**Published:** 2025-07-30

**Authors:** Z. J. Zhang, M. S. Ali, R. Hegde, R. H. Jugo, T. A. Zhang, S. H. Kurtzman

**Affiliations:** 1 Department of Surgery, Frank H Netter MD School of Medicine Waterbury Hospital, 64 Robbins St, Waterbury, CT 06708 USA; 2 Department of Colorectal Surgery Springfield, Springfield, MO USA; 3 Georgetown University, Washington, DC, USA

**Keywords:** Fistula-in-ano, Transsphincteric fistula, Modified TROPIS, Single-stage operation, Fecal incontinence

## Abstract

**Purpose:**

For treating complex transsphincteric fistula, a two-stage approach is usually administered: an initial seton placement followed by a sphincter-sparing procedure. However, success rates are not optimal. This study aimS to describe the modified transanal opening of the intersphincteric space (TROPIS), a single-staged procedure for managing transsphincteric fistula with or without concurrent anorectal abscess, and to compare its efficacy with the LIFT.

**Methods:**

Thirty-six patients who presented with mid-high transsphincteric fistula with or without associated anorectal abscess and consented to the procedure from 2020 to 2023 were managed with modified TROPIS. The primary outcome measures were recurrent fistulas and fecal continence. These results were compared with our previous study data of 24 patients who underwent LIFT procedure from 2011 to 2013.

**Results:**

Thirty-six patients received modified TROPIS; nine (25.0%) had an associated ischiorectal abscess. At the 8-month and 14-month follow-up, zero patients experienced fistula recurrence or fecal incontinence. In comparison with our previous study, 24 patients with transsphincteric fistula with or without associated abscess were treated with initial seton placement, then LIFT. With a follow-up range of 14–36 months, five (20.8%) patients presented with recurrent fistulas; no patients experienced fecal incontinence. These results were statistically significant.

**Conclusions:**

Our results reflect that modified TROPIS is a safe, simple, and effective procedure for treating patients with transsphincteric fistula with or without associated abscess. Patients healed with no fistula recurrence, which is significant in comparison with previous patients treated with LIFT. Modified TROPIS does not require an initial seton placement for managing transsphincteric fistula with associated abscess.

## Introduction

An anorectal fistula is the result of a chronic process when an abscess ruptures or drains into an epithelized tract that connects the abscess cavity with the skin. Around 15–35% of patients presenting with an anorectal abscess develop a fistula-in-ano, which occurs more commonly in adult males than females [1–4]. Surgical treatment of fistula-in-ano aims to manage the fistula tract while preserving the sphincter muscles. A transsphincteric fistula is the second most common type of fistula and occurs owing to a chronic abscess cavity in the ischiorectal space resulting in the infection progressing through the internal and external sphincters to the external skin. Despite multiple treatment modalities, the success rates have been variable for transsphincteric fistulas. The LIFT procedure first described in 2007 by Rojanasakul et al. initially demonstrated good outcomes [5]; however, it was associated with recurrence rates ranging from 6–28% when patients were followed for over 30 months [6]. Furthermore, if the patient presents with an associated anorectal abscess, an initial seton placement operation is required, and the LIFT procedure may then be administered 6–8 weeks afterward, making the treatment a lengthy two-stage process. Other treatment options that have resulted in inconsistent healing include endorectal advancement flap with recurrences ranging from 0–40%, fibrin glue sealant with recurrences ranging from 14–69%, and fistula plug with recurrence rates as high as 71% [7]. A systematic review by Kontovounisios et al. showed success rates in 19 studies ranging from 51% to 94% for LIFT, from 20% to 100% for advancement flap procedures, from 0% to 86% for fibrin glue sealants, and from 14% to 100% for anal collagen plugs, all with no significant difference in success rates relative to the number of patients in each study [8].

We hypothesize that fistula recurrence may be traced back to persistent undrained collections with retained infected anal crypt gland. For instance, while the LIFT procedure ligates the fistula tract, the internal opening with infected gland remains, and intraluminal pressures have not been relieved. Therefore, we believe that optimal management of a transsphincteric fistula should include three components: (1) cauterizing the infected crypt gland to eliminate the source of the abscess formation, (2) widening and externalizing the internal opening to allow for healing via secondary intention, and (3) debriding the abscess cavity and fistula tract from the internal opening to the external opening.

We therefore propose the modified TROPIS; the original approach was first described by Pankaj Garg in 2017 [9]. First, the transsphincteric fistula and the intersphincteric groove are identified. Second, a fistulotomy (laying open the fistula tract) at the intersphincteric level is performed by incising a portion of the internal sphincter muscle while preserving the external sphincter muscles. Through this externalization of the internal opening, the infected gland may be eliminated and allowed to heal by secondary intention. Lastly, the fistula tract is debrided, and the external opening is widened. Since the modified TROPIS only incises a portion of the internal sphincter muscle and fully preserves the external sphincter muscle, continence risks should be similar to that of a fistulotomy generally used in the treatment of an intersphincteric fistula-in-ano.

The author and colleagues performed a retrospective study evaluating the healing outcomes of patients with transsphincteric fistulas, who were treated with either modified TROPIS or the LIFT procedure.

## Methods

### Study design

This is an Institutional Review Board-approved retrospective study at an academic teaching hospital. All 36 patients with confirmed mid or high transsphincteric fistula were thoroughly debriefed on the procedure and provided full informed consent to participate in the modified TROPIS study. The modified TROPIS procedures were performed from January 2020 to September 2023. These results were compared with our previous study data of 24 patients who received LIFT procedure and underwent operation from January 2011 to May 2013, which was presented at the 2015 ASCRS meeting [10].

### Patients

In our practice, patients are advised to maintain a clear liquid diet for 24 h prior to surgery and take one enema the morning of surgery. We also advise patients to discontinue all drugs that can prolong bleeding (e.g., coumadin, aspirin-containing medications, nonsteroidal anti-inflammatory drugs). Mechanical bowel preparation is not required. Postoperatively, a compound ointment was administered which contains 3% diclofenac, 1.5% lidocaine, 10% Flagyl, and 0.2% nitroglycerin. No antibiotics were required for this procedure. This was an outpatient 1-day surgery, and none of the patients required hospital admission during the perioperative period. The Wexner continence grading scale was used to assess for postoperative continence at 8 months and 14 months after surgery. Patients with Crohn’s disease were not included in this study.

### Operative technique for modified TROPIS

**Fig. 1:**
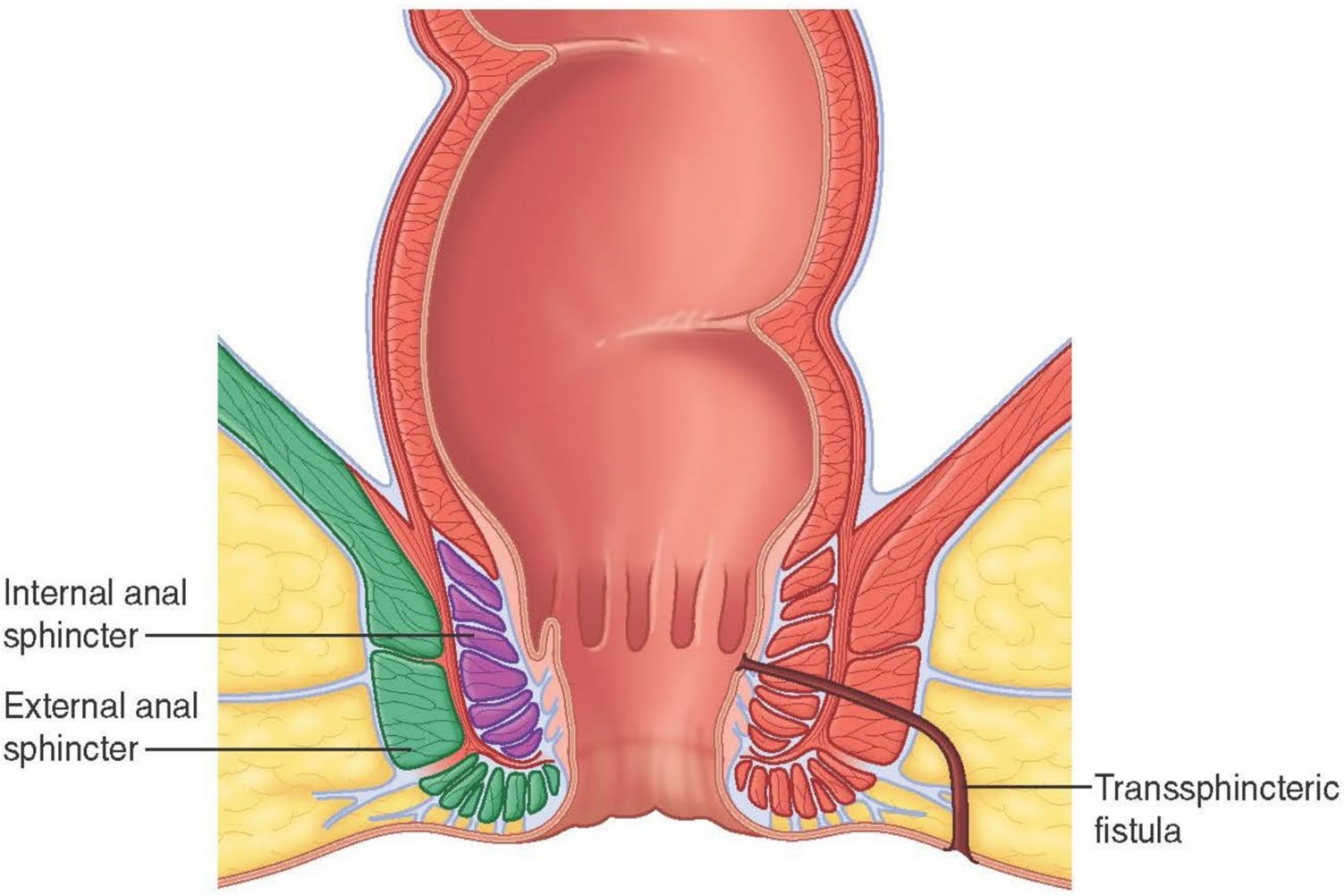
Anatomy of transsphincteric fistula

**Fig. 2:**
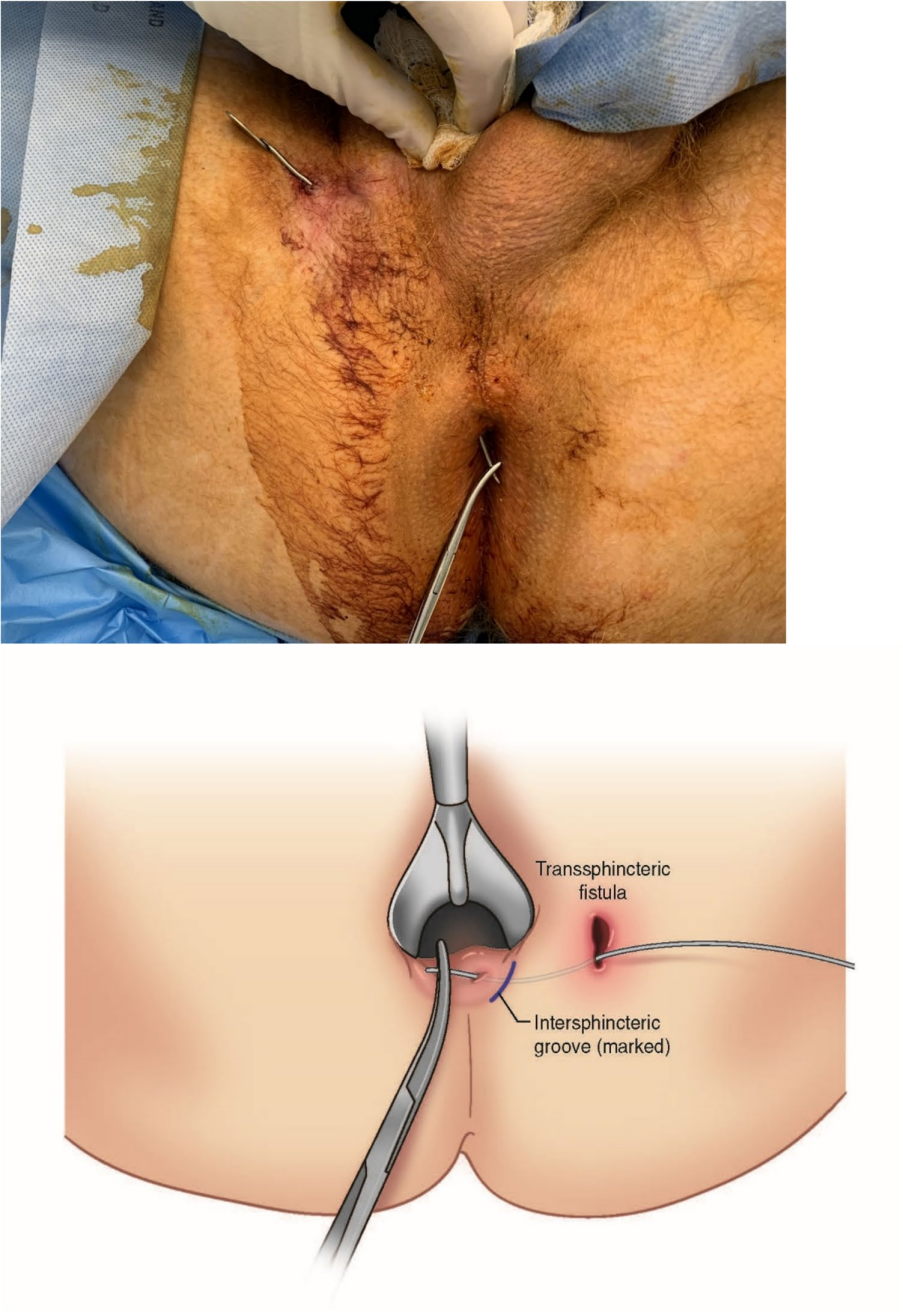
Lacrimal probe is passed through the fistula tract, and the intersphincteric groove is marked

**Fig. 3:**
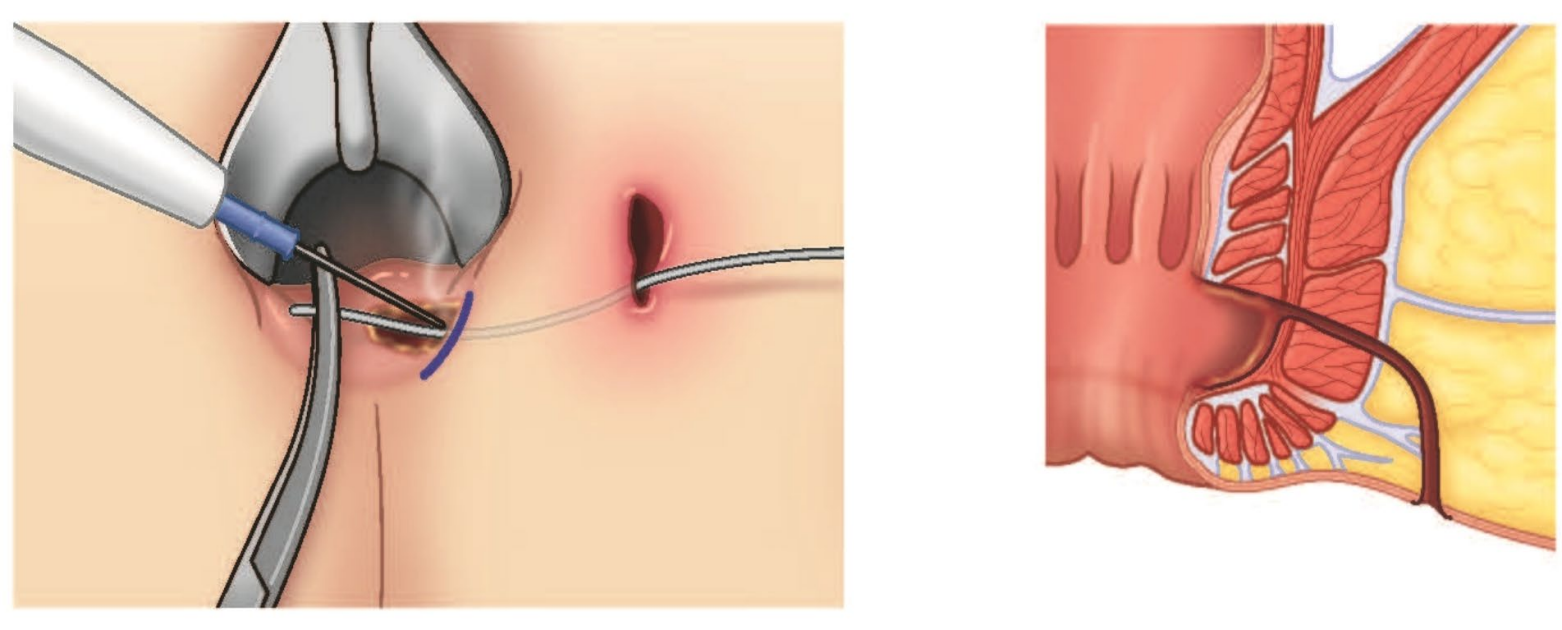
Modified TROPIS performed with cautery to divide a portion of the internal sphincter muscle

Firstly, the patient was given general anesthesia with laryngeal mask airways (LMA) and placed in a lithotomy position. Using a Hill–Ferguson lighted rectal retractor for visualization, the lacrimal probe was then placed in the external opening of the fistula tract and the internal opening of the fistula tract was identified to confirm the presence of a mid-high transsphincteric fistula (Fig. 1). The skin over the intersphincteric groove was palpated and marked (Fig. 2). The modified TROPIS was then performed by using electrocautery to lay open the fistula tract from the internal opening to the intersphincteric groove as depicted in Fig. 3. A portion of the internal sphincter muscle was cut, and the external sphincter muscle was fully preserved. Subsequently, the internal opening was externalized to the anal verge (Fig. 3). This procedure is similar to a regular fistulotomy for intersphincteric fistula.

**Fig. 4:**
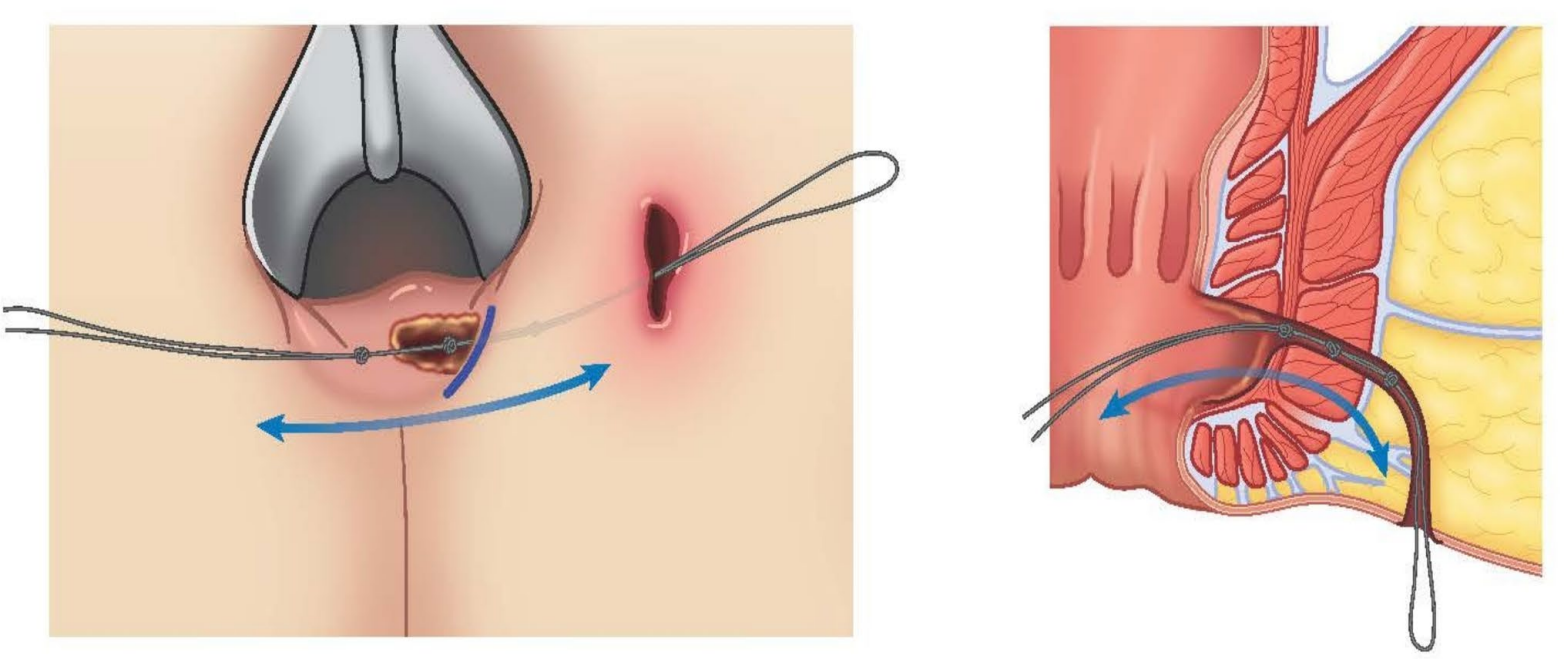
Knotted 0-Vicryl suture passed back and forth through fistula tract to debride the fistula tract

The lacrimal probe was then retracted, and the fistula tract was debrided with the back of a knife handle at the fistulotomy site and a knotted 0-Vicryl suture using a back-and-forth motion to clear all granulation tissue and to remove the epithelialized portion of the tract (Fig. 4). The external opening was left patent. The abscess cavity was debrided. The wound was cleaned, dried, and covered with Bacitracin ointment and 4 × 4 dry gauze.

### Postoperative care, patient follow-up, and outcomes

This is a retrospective case series. Postoperatively, patients were recommended for self-care of wounds with Sitz-baths for 15 min a few times a day as needed for assistance in pain control. No antibiotics were involved during the management of these patients. Patients were discharged according to a 1-day procedure. The data collected during the operation included type of fistula and location of fistula tract openings, and clinical follow-up at 2 weeks, 4 weeks, 8 months, and 14 months was performed to assess for any recurrences and fecal incontinence.

### Statistical analysis

Fisher’s exact test was used for biostatistical analysis to compare recurrent fistula rates between the 36 patients who underwent modified TROPIS and the 24 patients who underwent the LIFT procedure.

## Results

The ages of the patients who received modified TROPIS ranged from 21–68 years with a median of 49.5 years; 31 were male, and 5 were female. Not including routine postoperative follow-up visits, two additional follow-up visits were conducted: one at 8 months and another at 14 months. All 36 patients presented with a transsphincteric fistula. Two patients had a previous LIFT procedure, which recurred as a transsphincteric fistula. Four patients had seton placement prior to referral to our institution. Nine patients presented with an associated ischiorectal abscess identified during the operation. Comorbidities included five patients who were obese, and one patient who had ulcerative colitis (UC). For the patient with UC, modified TROPIS was performed after total abdominal colectomy with end ileostomy. Afterwards, he completed J-pouch surgery: completion proctectomy with ileal pouch-anal anastomosis and diverting loop ileostomy (DLI), and subsequently, the DLI was closed. This patient successfully maintained fecal continence. During postoperative follow-up (2 weeks and 4 weeks), patients usually experience mild anal pain and drainage. During the 8 month and 14 month postoperative follow-up, there were no fistula recurrences, persistent anal pain, or fecal incontinence (Table 1).

The ages of our previous study patients who received the LIFT procedure ranged from 23 to 83 years with a median of 47.5 years; 15 were male, and 9 were female. Median follow-up was 12.5 months with a range of 2–34 months. Among the 24 patients who had transsphincteric fistula, 2 had two fistulas and 2 had previous surgery. Of the previous surgeries, one had an endorectal advancement flap and one had a fistula plug. All 24 patients underwent seton placement prior to the LIFT procedure. Comorbidities included four patients who were obese, five patients with diabetes mellitus, and four patients who were smokers. Nineteen (91.7%) patients underwent successful closure without recurrence or failure. Five patients (20.8%) presented with recurrent fistulas after the LIFT procedure, two of which recurred as intersphincteric fistula, and three recurred as transsphincteric fistula. The two patients with intersphincteric fistula recurrence required a fistulotomy. Two of three patients with transsphincteric fistula recurrence then underwent modified TROPIS, and the third patient underwent fistula plug at another institution. Median healing time was 4 weeks with a range of 2–12 weeks. Median time to failure/recurrence was 12 weeks. Fecal continence was maintained in all 24 patients (Table 1).

It is significant to note that one patient who initially presented with transsphincteric fistula with associated ischiorectal abscess experienced a postoperative abscess recurrence after the modified TROPIS procedure. Upon examination under anesthesia, we found that the abscess recurred owing to early closure of the external opening in the skin; however, there was no recurrent fistula. We performed incision and drainage of the abscess with a mushroom catheter placement (Teleflex Pezzer Catheter 10FR), which was removed during the 2-week follow-up, and the abscess was completely healed. Therefore, this presents a potential amendment to the modified TROPIS procedure depending on the size of the associated ischiorectal abscess of the patient: specifically, for especially large associated abscesses, a mushroom catheter placement is recommended during the procedure, which would be removed at the 2 week follow-up.

**Table 1:** Comparison of patients with fistula recurrence, associated abscess, and other patient factors in the modified TROPIS and LIFT study groups

Study group	Associated abscess	Prior seton	History of UC	Prior LIFT	Fecal incontinence	Fistula recurrence (%) ^*^
Modified TROPIS *n* = 36	9	4	1	2	0	0
LIFT *n* = 24	0	24	0	N/A	0	5 (21%)

*UC* ulcerative colitis

^*^P value = 0.02 (Fisher’s exact test)

## Discussion and conclusions

The idea for this procedure in treating transsphincteric fistula came about owing to the high rate of transsphincteric fistula recurring as intersphincteric fistula after treatment of the initial transsphincteric fistula with the LIFT procedure [11]. The recurrent intersphincteric fistula would then be treated with a simple fistulotomy [11, 12]. We therefore hypothesized that, if the transsphincteric fistula is initially treated with a procedure similar to the simple fistulotomy and the infection source is addressed, this could possibly both heal the fistula and prevent fistula recurrence.

We believe that a significant factor contributing to the high rate of fistula recurrence in patients after the LIFT procedure is persistent undrained collections with infected gland, which are not relieved by the procedure as the small internal opening remains despite the ligated tract. Therefore, we proposed the modified TROPIS, which not only aims to drain abscesses and eliminated the infected gland to prevent recurrence, but also addresses other key components in fully managing a transsphincteric fistula. The idea for the modified TROPIS came about owing to the very strict indications for TROPIS and relatively large incision in the rectum, causing bleeding. The modified TROPIS (1) eliminates the source of abscess formation by cauterizing the infected crypt gland, (2) allows for drainage of the abscess by widening the internal opening, and (3) promotes healing of the fistula tract through healing by secondary intention. Although a portion of the internal sphincter muscle is cut, the external sphincter muscle is left intact and continence is not affected, which is reflected by our findings.

The LIFT procedure was reported as a sphincter-sparing procedure for transsphincteric fistulas, first described in 2007 [5]. The procedure is based on the secure closure of the internal opening at intersphincteric plane. Although the LIFT procedure demonstrated high primary healing rate (94.4%) [5], two meta-analyses report that the LIFT procedure achieved fistula healing in 61–94% of patients in 4–8 weeks, with low morbidity (14%) and rare fecal incontinence (1.4%) [13, 14]. Although the LIFT procedure demonstrated decreased rates of incontinence in comparison with the advancement flap [15], it is usually a two-stage process that still results in recurrent fistulas. In comparison, a modified TROPIS is a single-stage procedure that can be performed even when the patient presents with a concurrent abscess during the operation. Our data showed that 9/36 (25.0%) patients had an associated abscess identified during the modified TROPIS, and these patients did not develop fistula recurrence.

With similar ideas, fistulectomy with immediate sphincter reconstruction is another procedure that has been described for managing transsphincteric fistulas [16]. Although Fakhrolsadat et al. demonstrated success rates upward to 98.8%, there was one patient in which there was a recurrence, and incontinence rates were upward of 8% [16]. A modified TROPIS has the added benefit of not requiring any incision of the external sphincter muscles and avoids the large sphincter reconstruction that can result in fecal incontinence and potential recurrences.

At our institution, the modified TROPIS is an outpatient procedure. Average Operating Room (OR) procedure times are roughly 17 min, and the patient’s pain is well controlled to go home with wound care instructions. There are no antibiotics administered and no preoperative seton, which may decrease costs for the patient and the hospital. The simplicity and reliability of a modified TROPIS with good initial results in our study are promising. Larger studies should be considered to increase the generalizability and confirm the efficacy of our findings. The modified TROPIS may be performed for low, mid, and high transsphincteric fistula, where it provides maximum physical convenience for high transsphincteric fistula in obese patients. It should be noted that, as the modified TROPIS should have a similar incontinence risk to that of a fistulotomy as it incises a similar amount of the internal sphincter muscle, this procedure may not be recommended for populations who present with baseline mild fecal incontinence. We have learned that, for patients who present with concurrent large associated abscess, modified TROPIS may still be performed, but a mushroom catheter must be placed to prevent closure of the external opening prior to complete abscess drainage and healing. This would subsequently minimize postoperative abscess reformation.

In conclusion, the modified TROPIS is a safe, simple, and effective procedure for treating patients with transsphincteric fistula-in-ano with or without concurrent abscess. Our patients healed with no fistula recurrence or fecal incontinence, which is remarkable in comparison with previous patients treated with LIFT procedure. Modified TROPIS does not require an initial seton placement unlike previous operations for managing transsphincteric fistula with associated abscess. Furthermore, this procedure is an outpatient 1-day surgery, allowing for decreased operating room time and hospital costs. Finally, modified TROPIS may be performed for low, mid, and high transsphincteric fistula, where it provides maximum convenience for high fistula in obese patients. Further studies should be considered to strengthen the generalizability of our results.

## Data Availability

No datasets were generated or analyzed during the current study.
